# The Effect of Stress on a Personal Identification System Based on Electroencephalographic Signals

**DOI:** 10.3390/s24134167

**Published:** 2024-06-27

**Authors:** Eman A. Abdel-Ghaffar, May Salama

**Affiliations:** Electrical Engineering Department, Faculty of Engineering Shoubra, Benha University, Cairo 11629, Egypt; may.mohamed@feng.bu.edu.eg

**Keywords:** electroencephalogram (EEG), biometric systems, human affective state, stress

## Abstract

Personal identification systems based on electroencephalographic (EEG) signals have their own strengths and limitations. The stability of EEG signals strongly affects such systems. The human emotional state is one of the important factors that affects EEG signals’ stability. Stress is a major emotional state that affects individuals’ capability to perform day-to-day tasks. The main objective of this work is to study the effect of mental and emotional stress on such systems. Two experiments have been performed. In the first, we used hand-crafted features (time domain, frequency domain, and non-linear features), followed by a machine learning classifier. In the second, raw EEG signals were used as an input for the deep learning approaches. Different types of mental and emotional stress have been examined using two datasets, SAM 40 and DEAP. The proposed experiments proved that performing enrollment in a relaxed or calm state and identification in a stressed state have a negative effect on the identification system’s performance. The best achieved accuracy for the DEAP dataset was 99.67% in the calm state and 96.67% in the stressed state. For the SAM 40 dataset, the best accuracy was 99.67%, 93.33%, 92.5%, and 91.67% for the relaxed state and stress caused by identifying mirror images, the Stroop color-word test, and solving arithmetic operations, respectively.

## 1. Introduction

In recent years, growing interest has been devoted to studying the strength of using brain waves as a biometric modality. Brain waves offer a high degree of uniqueness, permanence, and universality and are very difficult to spoof. Existing EEG-based personal identification systems either use features extracted from EEG signals followed by a machine learning classifier or use raw EEG signals as the input to deep learning models.

A biometrics authentication system comprises verification and identification processes. Verification is the process of answering the following question: “Is this person who he claims he is?”. In the verification process, the system receives the biometric data from a participant along with his/her claimed identity. The system compares the biometric data to the data of only that participant in the database. The verification system is a 1:1 matching system [1,2]. When evaluating the performance of the verification system, the equal error rate (EER) is one of the most commonly used metrics. The EER is the location on a receiver operating characteristic curve (ROC) where the false acceptance rate and false rejection rate are equal. The lower the EER value, the higher the accuracy of the verification system is.

Identification (recognition) is the process of answering the following question: “Who is this person?”. The term identification and the term recognition have the same meaning and are used interchangeably. In the identification process, the system receives the biometric data from an unknown participant and compares them to the data of all the participants in the database. The identification system is a 1:*N* matching system, where *N* is the total number of participants in the system database [1,2]. The identification process takes longer than the verification process, as the system compares the reference data against all subjects to find a match. The correct recognition rate (CRR) is one of the most commonly used metrics for evaluating the identification system’s performance. Throughout this work, the terms CRR, identification accuracy, and recognition accuracy are used interchangeably.

In personal identification systems based on feature extraction, the feature selection step simplifies the model, prevents over-fitting, and reduces the training time. Selected features can either be in the time domain, frequency domain, or time–frequency domain. Then, the feature vector is fed into a machine learning classifier such as a support vector machine (SVM), k-nearest neighbors (KNN), Linear Discriminant Analysis (LDA), or random forest (RF). In [3], Bak and Jeong proposed an EEG motor imagery (MI) methodology for user identification. They extracted four features related to MI and compared the accuracy for recognizing users using Gaussian Naïve Bayes (GNB) and support vector machine (SVM). They achieved a user identification accuracy of 97.47% and 98.97% using GNB and the SVM, respectively. Wijayanto et al. in [4] proposed a biometric identification system based on EEG signals, and they used the Hjorth Descriptor; the highest achieved accuracy was 100%. In [5], Thomas and Vinod used the Mahalanobis distance as a classifier in performing person authentication based on EEG signals during the resting state with both eyes open (EO) and eyes closed (EC) using power spectral density and sample entropy; their system achieved a genuine accept rate (GAR) of 99.7% and 98.6% for EO and EC, respectively. Piciucco et al. [6] introduced a personal identification system based on steady-state visually evoked potentials (SSVEPs). EEG responses to SSVEP stimuli were recorded. Autoregressive (AR) and Mel frequency cepstral coefficients (MFCCs) were used as features and the Manhattan distance as a classifier. The best accuracies they achieved were 96% and 94.53% for MFCCs and AR, respectively. Monsy in [7] examined personal authentication from EEG signals in the resting state using frequency-weighted power (FWP) and achieved an equal error rate (EER) of 0.0039 from the EC resting state. In [8], Abdelghaffar et al. offered a personal authentication and cryptographic key-generation system based on EEG signals. In the proposed system, they used EEG signals to generate different cryptographic keys with different lengths. They represented multichannel EEG signals as points on a Riemannian manifold and used the Reed–Solomon (RS) coder for error correction. The system was tested using three datasets, AMIGOS, DEAP, and SEED, achieving an accuracy of 96.23%, 98.85%, and 99.89%, respectively. In [9], Tatar used statistical methods to generate 15 features from 64 channel EEG signals of 96 subjects. The selected feature vector was fed as an input to the DNN model and traditional ML classifiers. His best average achieved accuracy was 100% using both random forest and DNN classifiers.

In personal identification systems based on deep learning models, the DL models automatically learn complex features from raw EEG data without using hand-crafted features. Lai et al. in [10] used resting state eyes open (REO) and resting state eyes closed (REC) EEG signals. They achieved a validation accuracy and test accuracy of 83.21% and 79.08%, respectively. They arranged the raw EEG signals in the form of a matrix and used it as an input to a five-layer CNN. In [11], Chu et al. used single-channel resting state EEG data from healthy control (HC) subjects, characteristics of individuals with high risk (CHR), and patients with schizophrenia (FES). They proposed a modified deep learning architecture to automatically extract individual’s unique features. There proposed system achieved a classification accuracy of 81.6% for CHR individuals, 96.7% for clinically stable FES, and 99.2% for HC subjects. In [12], Das et al. proposed an unsupervised autoencoder CNN model for learning sparse EEG features. Their system was tested on motor imagery EEG (MI-EEG) signals from 109 subjects. The highest recognition rate they achieved for task-based identification was 87.60% and 99.89% for the resting state. In [13], Mao et al. used raw EEG signals from 100 subjects collected from a driving fatigue experiment to train a CNN model. They concluded that deep learning solutions are applicable to real-life EEG-based biometric identification, achieving a 97% accuracy. Chen et al. in [14] fed raw EEG signals into a new CNN with a global spatial and local temporal filter (GSLT-CNN). Their system achieved 96% recognition accuracy on a dataset containing 157 subjects performing different tasks. Fidas et al. and Biradar et al. in [15,16] offered an extensive survey on the use of EEG signals for building biometric authentication systems.

Mental stress is a major problem that affects the individual’s capability to perform day-to-day life tasks. Stress has a direct effect leading to several diseases including depression, cardiovascular disease, stroke, and cognitive problems [17]. Also, stress is considered as a contributing factor to several health problems including eating habits, sleeping problems, and skin conditions [18]. Mental stress assessment is a challenging problem as each person experiences stress differently [19]. The accuracy of detecting mental stress depends on the stress assessment method. Self-reported questionnaires are the most popular stress assessment methods [20]. However, this method is subjective and suffers from inaccurate self-report stress ratings. Another stress assessment method is to use physiological measures such as electrodermal activity (EDA), electroencephalograph (EEG), electromyogram (EMG), pupil diameter, and blood pressure [21].

EEG signals contain rich information about mental and emotional states [22,23,24,25]. Using EEG signals in stress detection has been extensively studied. Patel et al. in [26] used time and frequency domain features to train DL algorithms to identify emotional stress. They examined different DL models and found that CONVlD + BiLSTM provided the highest detection accuracy. In [27], Wen and Mohd Aris detected mental stress at various levels with 98% accuracy by using a hybrid approach for classification using k-means clustering and SVM. In [28], Fu et al. captured invariant and discriminative features from raw EEG signals using a deep neural network that combines a convolutional neural network (CNN) and symmetric deep convolutional adversarial network (SDCAN). Their proposed network achieved accuracies of 87.62% and 81.45% on the classification of four and five stress stages, respectively. In [29], Roy et al. applied an automatic feature extraction and classification model to 14-channel EEG signals, in order to efficiently detect psychological stress. First, they used Discrete wavelet transform (DWT) to decompose the EEG signals into different frequency bands. A CNN was deployed for automatic feature extraction, then bidirectional long short-term memory (BiLSTM) and two layers of a gated recurrent unit (GRU) were used for stress level classification. Katmah et al. in [30] offered a complete review on mental stress assessment methods using EEG signals.

Most of the existing studies focus on developing feature extraction and classification algorithms for either using EEG as a biometric modality or detecting and classifying different stages of stress. Biometric systems based on EEG signals are proven to be strong, but such systems suffer from some common limitations. The stability of EEG signals strongly affects such systems. Several factors affect EEG signals including performing different mental tasks, variation in emotional states, and recording in temporally spaced sessions. Few studies have addressed the impact of emotions on the performance of EEG-based biometric systems. In [31], Arnau et al. studied the influence of different levels of valance and arousal (high and low) on a human identification system. They used seven different machine learning classifiers to classify a feature vector that consisted of three different features (power spectral density (PSD), Mel frequency cepstral coefficients (MFCCs), and autoregression reflection coefficient (ARRC)). They concluded that the system performance is much higher when the valance and arousal level in training and testing match, compared to when they differ. Nguyena et al. in [32] verified the influences of negative and positive emotions on an EEG-based cryptographic key-generation system. The EEG data were labeled as high valance, low valance, high arousal, and low arousal. The cryptographic key was generated from EEG data by first extracting features using a parametric spectral estimation technique, then error correction was performed using a quantization technique. They decided that different levels of valance and arousal have impacts on the performance of the system.

The main objective of this work is to examine the stability of the EEG signal as a biometric modality under stress in a personal identification system. In the proposed study, the user enrollment stage is performed in the relaxed or calm state, while user identification is performed in the stressed state. Four types of stress were examined: stress caused by performing a mental arithmetic task, stress caused by performing a mirror image recognition task, stress caused by performing the Stroop color-word test, and emotional stress caused by watching video clips. To the best of our knowledge, this is the first work to study the effect of different types of stress on a human identification system based on EEG signals.

The rest of the paper is organized as follows; Section 2 gives an overview of the datasets used in our study. In Section 3, we introduce our experiment. In Section 4, we discuss and summarize our results. In Section 5, we conclude our work.

## 2. Datasets

**Figure 1:**
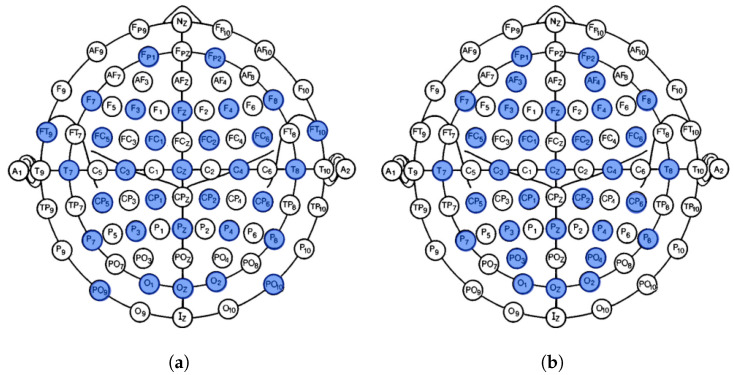
Electrode positioning: (**a**) SAM 40 dataset with 32 electrodes. (**b**) DEAP dataset with 32 electrodes.

The proposed study was performed using two publicly available datasets, SAM 40 [33] and DEAP [34]. Table 1 gives an overview of the two datasets, and their electrode positioning is shown in Figure 1.

### 2.1. SAM 40 Dataset

In the SAM 40 dataset [33], the EEG data were recorded from 40 subjects (26 male and 14 female, mean age 21.5 years). All 40 subjects were included in our study. The EEG data were acquired with a 32-channel Emotiv Epoc Flex gel kit t (Emotiv Inc., San Francisco, CA, USA) and sampled at 128 samples per second. The EEG signals were recorded from the subjects while performing various tasks such as solving arithmetic problems, the Stroop color-word test, the identification of symmetric mirror images, and a state of relaxation. Each individual task was carried out for 25 s. Three trials were recorded for each task. The EEG data were processed to remove artifacts. Each subject provided feedback on a scale of 1–10 depending on the stress levels he/she experienced during a particular task in a trial.

### 2.2. DEAP Dataset

In the DEAP dataset [34], the EEG and other physiological signals of 32 participants (16 male and 16 female, aged 19–37) were recorded while each of them watched 40 one-minute musical videos. The EEG signals were recorded using 32 electrodes placed according to the 10–20 international positioning system at a sampling rate of 512 Hz. The DEAP dataset has a pre-processed version in which, the electrooculography (EOG) artifacts were removed and the signals were down-sampled to 128 Hz and filtered from 4 to 45 Hz. Each observation (trial) was 63 s, in which the first 3 s were baseline signals. In this work we used the pre-processed version of the DEAP dataset, and the first 3 s were removed.

The DEAP dataset offers a quantitative description of the emotion state. Each participant was asked to offer a self-assessment of arousal, valance, dominance, and liking on a continuous 9-point scale after each trial. The calm and stress emotions were identified using valance and arousal scores using Equations (1) and (2) derived from [35,36,37]. By applying the rules for selecting the calm and stressed states from each participant trial, the results of 25 participants met the rules, but only 14 of them had a balanced number of calm and stress trials. Therefore, in our study, the rest of the DEAP analysis continued with the data of 14 participants (with the following participants IDs: 2, 4, 10, 11, 12, 13, 15, 16, 20, 21, 22, 25, 31, 32).

(1) $$\mathrm{Calm}=\left(\mathrm{arousal}<4\right)\cap \left(4<\mathrm{valance}<6\right)$$

(2) $$\mathrm{Stress}=\left(\mathrm{arousal}>5\right)\cap \left(\mathrm{valance}<3\right)$$

## 3. Experiment

In this work, we study the effect of stress on using EEG signals as a biometric trait in a personal identification system. Two different datasets were used for this purpose. In the DEAP dataset, participants offered a self-assessment of arousal, valance, dominance, and liking on a continuous 1–9-point scale. Calm and stress were identified using valance and arousal scores (see Equations (1) and (2)). Participants in the SAM 40 dataset labeled each trial as either relaxed or stressed, and three types of stress existed. To perform our experiments, for the DEAP dataset, enrollment was performed in the calm state, and identification was performed once in the calm state and again in the stressed state; for the SAM 40 dataset, enrollment was performed in the relaxed state, and identification was performed in the relaxed state, under stress caused by solving arithmetic problems, under stress caused by the Stroop color-word test, and under stress caused by identifying mirror images.

We performed two experiments. In the first, different sets of features (time domain, frequency domain, and non-linear features) were extracted from the EEG signals, and the classification was performed using the support vector machine (SVM) classifier. The SVM was chosen as the ML classifier as it offers good performance when the dataset is small, and it has been used in various EEG-based identification systems in the literature [3,9]. In the second experiment, raw EEG signals were used as the input to different deep learning (DL) models. The SAM 40 and DEAP datasets have a limited number of subjects, consisting of only 40 and 14 individuals, respectively. Also, each subject in those datasets has a limited number of trials. This limited amount of data poses a challenge, specially for deep learning techniques. In this work, we addressed this problem by dividing each trial into 5 s segments. Segments from the same trial shared the same label.

### 3.1. Feature Extraction

Features from different domains were extracted seeking better signal presentation, which could provide better personal identification performance. We used three types of features: frequency domain features using band power (FD-BP), time domain features using Hjorth parameters (TD-HPs), and non-linear features using Higuchi’s fractal dimension (NL-HFD). Classification was performed using the SVM.

**Figure 2:**
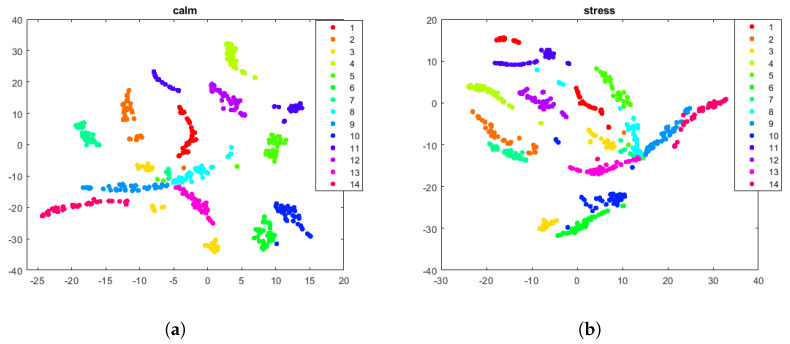
Band power feature for the 14 participants in the DEAP dataset. (**a**) Calm state. (**b**) Stress state. Visualization obtained through the t−SNE method using the Euclidean distance.

Band power (BP) is a frequency domain feature in which we calculate the power in different frequency bands (theta (4–8 Hz), alpha (8–12 Hz), beta (15–30 Hz), and gamma (30–45 Hz)). The delta frequency band (0–4 Hz) was excluded as the pre-processed version of the DEAP dataset is filtered from 4–45 Hz. The BP feature vector for each user in each 5 s segment consisted of 128 values (4 BP values X 32 electrodes). Figure 2 is a t-SNE figure that shows the BP feature for the 14 individuals in the DEAP dataset in the calm and stressed states.

The Hjorth parameters (HPs) are time domain features based on the variance of the derivatives of the EEG signal. The most commonly used HPs are activity, mobility, and complexity [38,39]. Activity represents the signal power, computed by calculating the variance of the signal. Mobility is the standard deviation of the first derivative of the EEG signal, divided by the standard deviation of the primary signal. Complexity gives an estimate of the bandwidth of the signal, and it is defined as the ratio of the mobility of the first derivative of the signal to the mobility of the signal (Equation (3)). The HP feature vector for each user in each 5 s segment consists of 96 values (3 HP values X 32 electrodes).

(3) $$\begin{array}{c} \begin{array}{c} \mathrm{Activity}=var(x(t)),\\ \mathrm{Mobility}=\sqrt{\frac{var(\frac{dx(t)}{dt})}{var(x(t))}},\\ \mathrm{Complexity}=\frac{\mathrm{Mobility}(\frac{dx(t)}{dt})}{\mathrm{Mobility}(x(t))} \end{array} \end{array}$$

where x(t) is the EEG signal recorded from a single electrode and var(x(t)) is the variance of the signal x(t).

To detect the hidden information contained in the EEG signals, the fractal dimension (FD) [40] is used. There are many methods that can be used to calculate the FD such as Katz’s, Higuchi’s, and Petrosian’s methods [41]. Higuchi’s method has proven to be the most accurate estimate of the FD [42,43,44]. Higuchi’s fractal dimension originated from chaos theory, and it can detect hidden information contained in biological time series regardless of the nature of the analyzed signal (deterministic or stochastic, stationary or non-stationary) [45]. In this work, we used the HFD to form a quantitative measure of the signal dynamics. The HFD feature vector for each user in each 5 s segment consisted of 32 values.

### 3.2. Raw EEG Signals

**Figure 3:**
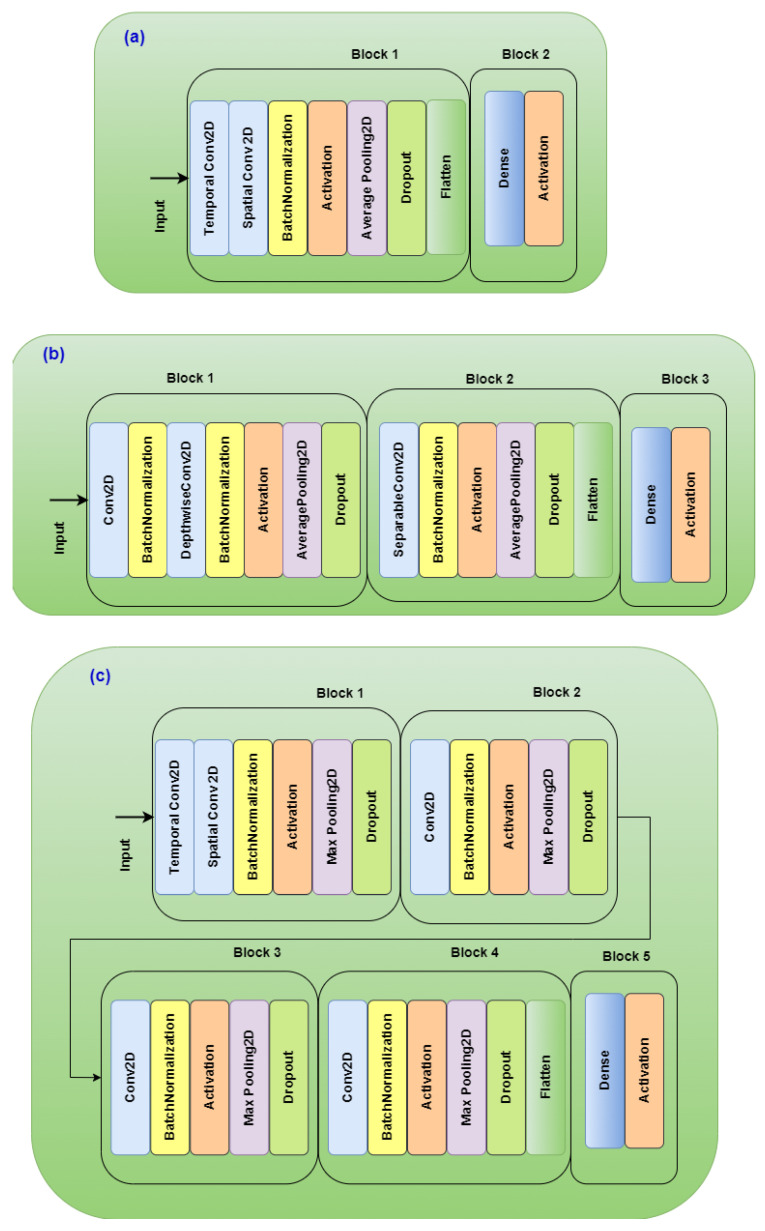
Network architectures. (**a**) Shallow CNN. (**b**) EEGNet. (**c**) Deep ConvNet.

Deep learning approaches are capable of automatically learning complex features from raw EEG data without using hand-crafted features. Several CNN models using raw EEG signals as inputs have been proposed [46,47,48,49,50]. In this work, three well-known EEG-based CNNs, Shallow ConvNet, EEGNet, and Deep ConvNet, were used. For the three networks, the softmax method is used in the classification layer, and the number of epochs was 300. Figure 3 shows the basic architecture of EEGNet, Shallow ConvNet, and Deep ConvNet.

The original Shallow ConvNet introduced in [46] used temporal convolutions of length (1, 25), a pool size of (1, 75), and strides of (1, 13) for EEG with a 250 Hz sampling frequency. Since the two datasets we used in our study (DEAP and SAM 40) have a 128 Hz sampling frequency, we halved all those values, and we used temporal convolutions of length (1, 13), a pool size of (1, 35), and strides of (1, 7).

EEGNet is one of the most commonly used networks for BCI applications [51]. It was originally introduced by Lawhern et al. in [47]. They introduced a compact CNN, which consists of two convolutional pooling blocks followed by a classification layer. In our study, we used EEGNet 8-2 with the following parameters: Kernel length 64, 8 temporal filters, and 2 spatial filters.

Deep ConvNet was first introduced in [46]. Its architecture consists of four convolutional pooling blocks followed by a dense classification layer. Deep ConvNet has the same initial architecture as Shallow ConvNet followed by three identical convolution blocks. In our work, we used Deep ConvNet with temporal convolutions of length (1, 5), a pool size of (1, 2), and strides of (1, 2).

## 4. Results and Discussion

The objective of our work is not to build an advanced personal identification system, but rather, to determine weather mental stress has an impact on the personal identification system’s performance. To achieve this goal, we performed two experiments. In the first, three types of features were extracted from raw EEG signals: frequency domain (BP), time domain (HP), and non-linear features (HFD). Then, classification was performed using the SVM classifier. Using the train–test split method in the sklearn library, the data were divided into 70% for training and 30% for testing. In the second, raw EEG signals (from 32 electrodes) were used as the inputs to three widely used EEG-based CNNs: Shallow ConvNet, Deep ConvNet, and EEGNet. In both experiments, 10-fold cross-validation was performed. The performance was evaluated using the accuracy and F-score metrics (Equation (4)). Accuracy here represents the correct recognition rate (CRR) metric in personal identification systems.

(4) $$\begin{array}{c} \begin{array}{c} \mathrm{Accuracy}=\frac{\mathrm{TP}+\mathrm{TN}}{\mathrm{TP}+\mathrm{FP}+\mathrm{FN}+\mathrm{TN}}\\ \mathrm{Precision}=\frac{\mathrm{TP}}{\mathrm{TP}+\mathrm{FP}}\\ \mathrm{Recall}=\frac{\mathrm{TP}}{\mathrm{TP}+\mathrm{FN}}\\ F\;-\;\mathrm{Score}=\frac{2\times (\mathrm{Recall}\times \mathrm{Precision})}{\mathrm{Recall}+\mathrm{Precision}} \end{array} \end{array}$$

where we have true positives as TP, true negatives as TN, false positives as FP, and false negatives as FN.

**Figure 4:**
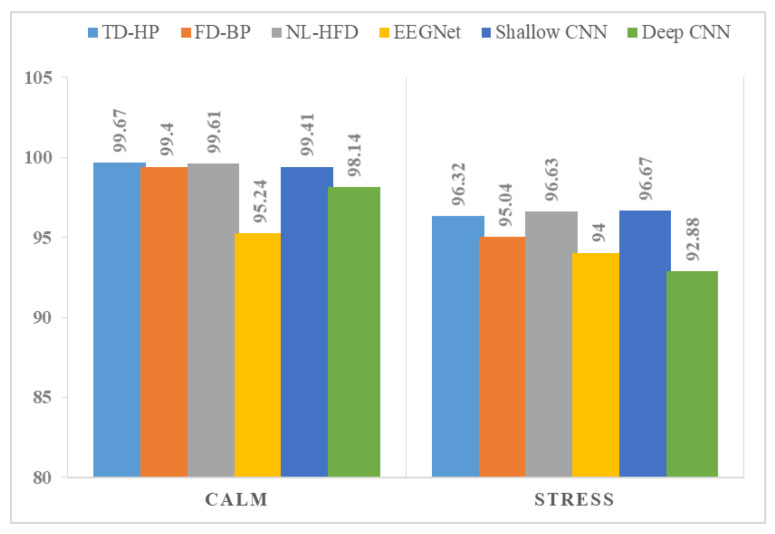
DEAP dataset difference in identification system accuracy in the calm and stressed states.

**Figure 5:**
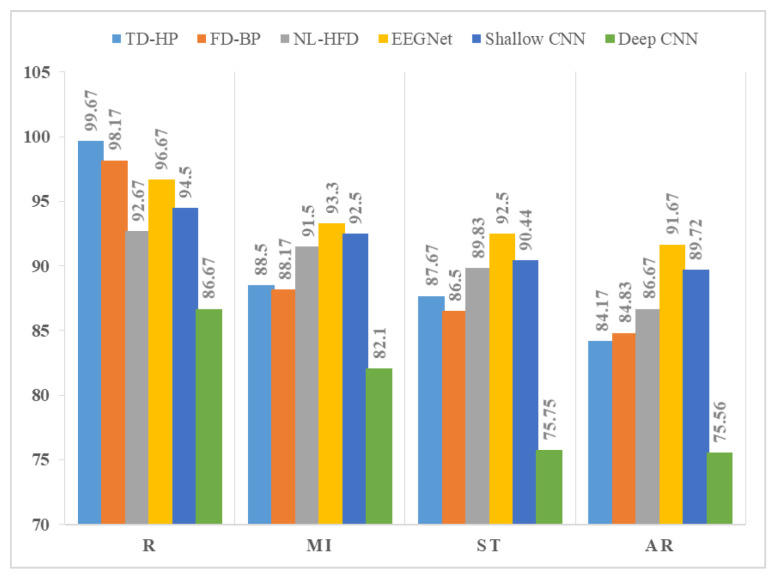
SAM 40 difference in identification system accuracy in the calm and stressed states. Relaxed state (R), solving arithmetic problems (AR), Stroop color-word test (ST), and identifying mirror image (MI) state.

Subject enrollment was performed in the calm or relaxed state, and identification was performed in the stressed state. Our study is not focused on building a more advanced EEG-based identification system; it is focused on using different emotions for the identification process. Yet, the achieved results are comparable to other existing systems. Examples of existing EEG-based personal identification systems in the literature are illustrated in Table 2. DEAP’s results are shown in Table 3, and SAM 40’s results are shown in Table 4. From these results we can see that the following:

- In both experiments, subject identification in the stressed state caused a reduction in the biometric system’s performance. The difference in identification system accuracy when enrollment was performed in the calm or relaxed state and identification was performed in the stressed state is clarified in Figure 4 and Figure 5.
- The identification system based on feature extraction showed the best results in the calm state, where the best accuracy was achieved by using time domain feature (Hjorth parameters (HPs)), while in the stressed state, non-linear features (Higuchi’s fractal dimension (HFD)) gave the best performance.
- The deep learning approaches were capable of learning features from raw EEG signals. The performances of Shallow ConvNet and EEGNet were very close to each other, while Deep ConvNet gave the worst performance. The biometric system based on the DL techniques was less affected by the change in human emotional states (relaxed or stressed) than the system based on hand-crafted features and the ML classifier.
- In the SAM 40 dataset, when testing different types of stress, it is clear that stress caused by identifying mirror images showed the least effect on biometric system performance. Stress caused by solving arithmetic operations and the Stroop color-word test showed the highest impact on system performance (the Stroop color-word test’s performance was slightly better than solving arithmetic operations).

## 5. Conclusions

In this article, we studied the effect of stress on an EEG-based human identification system. Four types of stress were examined: emotional stress caused by watching videos, stress caused by solving arithmetic problems, stress caused by the Stroop color-word test, and stress caused by identifying mirror images. Two experiments were performed. In the first experiment, we used hand-crafted features followed by an ML classifier; in the second experiment, the DL approaches were used. Performing enrollment in the relaxed or calm state and identification in the stress state affected the biometric system performance. The best achieved accuracy for the DEAP dataset was 99.67% in the calm state and 96.67% in the stressed state. For the SAM 40 dataset, the best achieved accuracy was 99.67%, 93.33%, 92.5%, and 91.67% for the relaxed state, stress caused by the identifying mirror images test, stress caused by the Stroop color-word test, and stress caused by solving arithmetic operations, respectively.

The identification system based on feature extraction showed the best results in the calm state, where the best accuracy was achieved by using the time domain feature, while in the stress state, non-linear features gave the best performance. The biometric system based on deep learning techniques was less affected by the change in human emotional states (relaxed or stressed) than the system based on hand-crafted features and the ML classifier. In the SAM 40 dataset, when testing different types of stress, it is clear that stress caused by identifying mirror images showed the least effect on biometric system performance. Stress caused by solving arithmetic operations and the Stroop color-word test showed the highest impact on system performance (the Stroop color-word test’s performance was slightly better than solving arithmetic operations).

## Figures and Tables

**Table 1:** An overview of the SAM 40 and DEAP datasets.

Item	SAM 40	DEAP
Recording Device	Emotiv Epoc Flex gel kit	Biosemi ActiveII
# of Subjects	40	32
Subjects’ Description	26 males, 14 females	16 males, 16 females
# of Electrodes	32	32
Sampling Rate	128 Hz	Originally 512 Hz, down-sampled to 128 Hz
Stimuli	Stroop color-word test, solving arithmetic questions, identification of symmetric mirror images, and a state of relaxation.	Different emotions caused by watching musical videos.
Trial Duration	25 s	63 s
Labels	Relaxed and three types of stress.	Continuous 9-point scale for arousal, valance, dominance, and liking.

**Table 2:** Examples of existing EEG-based personal identification systems in the literature.

Ref.	S, C	Dataset	Features	Classifier	Performance (CRR%)
[3]	52, 64	BMI	CSP, ERD/S, FFT, AR	SVM GNB	up to: 98.97 up to: 97.47
[4]	5, 4	SCD (photo stimuli)	HPs	NNT	up to: 100
[5]	109, 64	PhysioNet	PSD and SE	Mahalanobis distance	EO: 99.7 EC: 98.6
[6]	25, 19	SCD (SSVEPs)	MFCCs, AR	Manhattan distance	MFCCs: 95.87–96.0 AR: 91.47–94.53
[9]	96, 64	PhysioNet	15 features	DNN, SVM, DT, RF	DT: 98.63, RF: 100, KNN: 99.96, SVM: 99.91, DNN:100
[10]	109, 64	PhysioNet	Raw EEG signals	CNN	83.21
[11]	120, 64	SCD (rest state) HC, CHR, FES	Raw EEG signals	DNN	HC: 99.2, FES: 96.7, CHR: 81.6
[12]	109, 64	PhysioNet	Raw EEG signals	CNN	task: 87.60, non-task: 99.89
[13]	100,46	BCIT	Raw EEG signals	CNN	97
[14]	157, 64	X2 RSVP, XB Driving, DEAP, CT2WS RSVP	Raw EEG signals	CNN	96

**Note:** S: subjects; C: channels; SCD: self-collected data; CSP: common spatial pattern; ERD/S: event-related (de)synchronization; FFT: Fourier transform; AR: autoregressive; SVM: support vector machine; GNB: Gaussian Naïve Bayes; SSVEPs: steady-state visually evoked potentials; MFCCs: Mel frequency cepstral coefficients; HPs: Hjorth parameters; EO: eyes open; EC: eyes closed; PSD: power spectral density; SE: sample entropy; DNN: deep feature neural network; DT: decision tree; KNN: k-nearest neighbor; RF: random forest; HC: healthy controls; CHR: characteristics of individuals with high risk; FES: stable schizophrenia.

**Table 3:** DEAP results. The enrollment stage was performed in the calm state, while the identification stage was performed once in the calm state and another time in the stressed state (the best accuracies in the calm and stressed states are in bold).

Selected Features	Classifier	Calm		Stress	
		Acc.	F-Score	Acc.	F-Score
TD-HP	SVM	99.67	99	96.32	97
FD-BP	SVM	99.4	98	95.04	95
NL-HFD	SVM	99.61	98	96.63	96
Raw EEG	EEGNet	95.24	95	94	94
	Shallow ConvNet	99.41	99	96.67	97
	Deep ConvNet	98.41	97	92.88	92

**Table 4:** SAM 40 results. The enrollment stage was performed in the relaxed state, while the identification stage was performed in four different states: relaxed state (Relax), identifying mirror images (MI−Stress), Stroop color-word test (ST−Stress), and solving arithmetic problems (AR−Stress) (The best accuracies in the calm and stress states are in bold).

Selected Features		Relax		MI-Stress		ST-Stress		AR-Stress	
	Classifier	Acc.	F-Score	Acc.	F-Score	Acc.	F-Score	Acc.	F-Score
TD-HP	SVM	99.67	99	88.5	88	87.667	87	84.17	84
FD-BP	SVM	98.17	96	88.17	88	86.5	86	84.83	84
NL-HFD	SVM	92.67	91	91.5	91	89.83	90	86.67	86
Raw EEG	EEGNet	96.67	95	93.33	93	92.5	92	91.67	91
	Shallow ConvNet	94.5	94	92.5	93	90.44	89	89.72	89
	Deep ConvNet	86.67	85	82.1	82	75.75	75	75.56	74

## Data Availability

Publicly available datasets were analyzed in this study. These data can be found here: https://www.eecs.qmul.ac.uk/mmv/datasets/deap/index.html (accessed on 16 July 2019); https://doi.org/10.6084/m9.figshare.14562090.v1 (accessed on 12 May 2023).

## References

[1] Pradhan A., He J., Jiang N. (2022). Score, Rank, and Decision-Level Fusion Strategies of Multicode Electromyogram-Based Verification and Identification Biometrics. IEEE J. Biomed. Health Inform..

[2] Oloyede M.O., Hancke G.P. (2016). Unimodal and Multimodal Biometric Sensing Systems: A Review. IEEE Access.

[3] Bak S., Jeong J. (2023). User Biometric Identification Methodology via EEG-Based Motor Imagery Signals. IEEE Access.

[4] Wijayanto I., Hadiyoso S., Sekarningrum F.A. Biometric Identification Based on EEG Signal with Photo Stimuli using Hjorth Descriptor. Proceedings of the 8th International Conference on Information and Communication Technology (ICoICT).

[5] Thomas K.P., Vinod A.P. Biometric identification of persons using sample entropy features of EEG during rest state. Proceedings of the 2016 IEEE International Conference on Systems, Man, and Cybernetics (SMC).

[6] Piciucco E., Maiorana E., Falzon O., Camilleri K.P., Campisi P. Steady-State Visual Evoked Potentials for EEG-Based Biometric Identification. Proceedings of the 2017 International Conference of the Biometrics Special Interest Group (BIOSIG).

[7] Monsy J.C., Vinod A.P. (2020). EEG-based biometric identification using frequency-weighted power feature. IET Biom..

[8] Abdel-Ghaffar E.A., Daoudi M. (2023). Personal authentication and cryptographic key generation based on electroencephalographic signals. J. King Saud Univ.-Comput. Inf. Sci..

[9] Tatar A.B. (2023). Biometric identification system using EEG signals. Neural Comput. Appl..

[10] Lai C.Q., Ibrahim H., Abdullah M.Z., Abdullah J.M., Suandi S.A., Azman A. (2019). Arrangements of Resting State Electroencephalography as the Input to Convolutional Neural Network for Biometric Identification. Comput. Intell. Neurosci..

[11] Chu L., Qiu R., Liu H., Ling Z., Zhang T., Wang J. (2018). Individual Recognition in Schizophrenia using Deep Learning Methods with Random Forest and Voting Classifiers: Insights from Resting State EEG Streams. arXiv.

[12] Das B.B., Ram S.K., Babu K.S., Mohapatra R.K., Mohanty S.P. (2024). Person identification using autoencoder-CNN approach with multitask-based EEG biometric. Multimed. Tools Appl..

[13] Mao Z., Yao W.X., Huang Y. EEG-based biometric identification with deep learning. Proceedings of the 8th International IEEE/EMBS Conference on Neural Engineering (NER).

[14] Chen J.X., Mao Z.J., Yao W.X., Huang Y.F. (2020). EEG-based biometric identification with convolutional neural network. Multimed. Tools Appl..

[15] Fidas C.A., Lyras D. (2023). A Review of EEG-Based User Authentication: Trends and Future Research Directions. IEEE Access.

[16] Biradar S.D., Nalbalwar S.L., Deosarkar S.B. Biometric Security using EEG Signal Processing—Acquisition, Representation and Classification Approaches. Proceedings of the 2022 IEEE International Conference on Distributed Computing and Electrical Circuits and Electronics (ICDCECE).

[17] O’Connor D.B., Thayer J.F., Vedhara K. (2021). Stress and health: A review of psychobiological processes. Annu. Rev. Psychol..

[18] Thoits P.A. (2010). Stress and health: Major findings and policy implications. J. Health Soc. Behav..

[19] Hou X., Liu Y., Sourina O., Tan Y.R.E., Wang L., Mueller-Wittig W. EEG based stress monitoring. Proceedings of the 2015 IEEE International Conference on Systems, Man, and Cybernetics.

[20] Monroe S.M. (2008). Modern approaches to conceptualizing and measuring human life stress. Annu. Rev. Clin. Psychol..

[21] Giannakakis G., Grigoriadis D., Giannakaki K., Simantiraki O., Roniotis A., Tsiknakis M. (2019). Review on psychological stress detection using biosignals. IEEE Trans. Affect. Comput..

[22] She Q., Zhang C., Fang F., Ma Y., Zhang Y. (2023). Multisource Associate Domain Adaptation for Cross-Subject and Cross-Session EEG Emotion Recognition. IEEE Trans. Instrum. Meas..

[23] Li T., Fu B., Wu Z., Liu Y. (2023). EEG-Based Emotion Recognition Using Spatial-Temporal-Connective Features via Multi-Scale CNN. IEEE Access.

[24] Abdel-Ghaffar E.A., Wu Y., Daoudi M. (2022). Subject-Dependent Emotion Recognition System Based on Multidimensional Electroencephalographic Signals: A Riemannian Geometry Approach. IEEE Access.

[25] Abdel-Ghaffar E.A., Daoudi M. Emotion Recognition from Multidimensional Electroencephalographic Signals on the Manifold of Symmetric Positive Definite Matrices. Proceedings of the 2020 IEEE Conference on Multimedia Information Processing and Retrieval (MIPR).

[26] Patel A., Nariani D., Rai A. Mental Stress Detection using EEG and Recurrent Deep Learning. Proceedings of the 2023 IEEE Applied Sensing Conference (APSCON).

[27] Wen T.Y., Mohd A., Siti A. (2022). Hybrid Approach of EEG Stress Level Classification Using K-Means Clustering and Support Vector Machine. IEEE Access.

[28] Fu R., Chen Y., Huang Y., Chen S., Duan F., Li J., Wu J., Jiang D., Gao J., Gu J. (2022). Symmetric Convolutional and Adversarial Neural Network Enables Improved Mental Stress Classification From EEG. IEEE Trans. Neural Syst. Rehabil. Eng..

[29] Roy B., Malviya L., Kumar R., Mal S., Kumar A., Bhowmik T., Hu J.W. (2023). Hybrid Deep Learning Approach for Stress Detection Using Decomposed EEG Signals. Diagnostics.

[30] Rateb K., Fares A.S., Usman T., Fabio B., Fadwa A.M., Hasan A.N. (2021). A Review on Mental Stress Assessment Methods Using EEG Signals. Sensors.

[31] Arnau-González P., Arevalillo-Herráez M., Katsigiannis S., Ramzan N. (2021). On the Influence of Affect in EEG-Based Subject Identification. IEEE Trans. Affect. Comput..

[32] Dang N., Dat T., Dharmendra S., Wanli M. (2018). Emotional Influences on Cryptographic Key Generation Systems using EEG signals. Procedia Comput. Sci..

[33] Ghosh R., Deb N., Sengupta K., Phukan A., Choudhury N., Kashyap S., Phadikar S., Saha R., Das P., Sinha N. (2022). SAM 40: Dataset of 40 subject EEG recordings to monitor the induced-stress while performing Stroop color-word test, arithmetic task, and mirror image recognition task. Data Brief.

[34] Koelstra S., Muhl C., Soleymani M., Lee J., Yazdani A., Ebrahimi T., Pun T., Nijholt A., Patras I. (2012). DEAP: A Database for Emotion Analysis; Using Physiological Signals. IEEE Trans. Affect. Comput..

[35] Hasan M.J., Kim J.M. (2019). A Hybrid Feature Pool-Based Emotional Stress State Detection Algorithm Using EEG Signals. Brain Sci..

[36] Shon D., Im K., Park J.H., Lim D.S., Jang B., Kim J.M. (2018). Emotional Stress State Detection Using Genetic Algorithm-Based Feature Selection on EEG Signals. Int. J. Environ. Res. Public Health.

[37] Hag A., Handayani D., Altalhi M., Pillai T., Mantoro T., Kit M.H., Al-Shargie F. (2021). Enhancing EEG-Based Mental Stress State Recognition Using an Improved Hybrid Feature Selection Algorithm. Sensors.

[38] Grover C., Turk N. (2020). Rolling Element Bearing Fault Diagnosis using Empirical Mode Decomposition and Hjorth Parameters. Procedia Comput. Sci..

[39] Mehmood R.M., Bilal M., Vimal S., Lee S.W. (2022). EEG-based affective state recognition from human brain signals by using Hjorth-activity. Measurement.

[40] Raghavendra B.S., Dutt N.D. (2010). Signal characterization using fractal dimension. Fractals.

[41] García-Martínez B., Martínez-Rodrigo A., Alcaraz R., Fernández-Caballero A. (2021). A Review on Nonlinear Methods Using Electroencephalographic Recordings for Emotion Recognition. IEEE Trans. Affect. Comput..

[42] Raghavendra B.S., Dutt N.D., Halahalli H.N., John J.P. (2009). Complexity analysis of EEG in patients with schizophrenia using fractal dimension. Physiol. Meas..

[43] Esteller R., Vachtsevanos G., Echauz J., Litt B. (2001). A comparison of waveform fractal dimension algorithms. IEEE Trans. Circuits Syst. I Fundam. Theory Appl..

[44] Gladun K.V. (2021). Higuchi Fractal Dimension as a Method for Assessing Response to Sound Stimuli in Patients with Diffuse Axonal Brain Injury. Sovrem Tekhnol. Med..

[45] Kesić S., Spasić S.Z. (2016). Application of Higuchi’s fractal dimension from basic to clinical neurophysiology: A review. Comput. Methods Programs Biomed..

[46] Schirrmeister R.T., Springenberg J.T., Fiederer L.D.J., Glasstetter M., Eggensperger K., Tangermann M., Hutter F., Burgard W., Ball T. (2017). Deep learning with convolutional neural networks for EEG decoding and visualization. Hum. Brain Mapp..

[47] Lawhern V.J., Solon A.J., Waytowich N.R., Gordon S.M., Hung C.P., Lance B.J. (2018). EEGNet: A compact convolutional neural network for EEG-based brain—computer interfaces. J. Neural Eng..

[48] Yang J., Ma Z., Wang J., Fu Y. (2020). A Novel Deep Learning Scheme for Motor Imagery EEG Decoding Based on Spatial Representation Fusion. IEEE Access.

[49] Dai G., Zhou J., Huang J., Wang N. (2020). HS-CNN: A CNN with hybrid convolution scale for EEG motor imagery classification. J. Neural Eng..

[50] Köllőd C.M., Adolf A., Iván K., Márton G., Ulbert I. (2023). Deep Comparisons of Neural Networks from the EEGNet Family. Electronics.

[51] Liang X., Liu Y., Yu Y., Liu K., Liu Y., Zhou Z. (2023). Convolutional Neural Network with a Topographic Representation Module for EEG-Based Brain Computer Interfaces. Brain Sci..

